# Loss of *ICP22* in HSV-1 Elicits Immune Infiltration and Maintains Stromal Keratitis Despite Reduced Primary and Latent Virus Infectivity

**DOI:** 10.1167/iovs.19-27701

**Published:** 2019-08

**Authors:** Harry H. Matundan, Ujjaldeep Jaggi, Shaohui Wang, Homayon Ghiasi

**Affiliations:** Center for Neurobiology and Vaccine Development, Ophthalmology Research, Department of Surgery, Cedars-Sinai Burns & Allen Research Institute, Los Angeles, California, United States

**Keywords:** antigen-presenting cells, cornea, immediate early genes, corneal scarring, virus replication

## Abstract

**Purpose:**

We previously have reported that *ICP22*, an immediate early gene of herpes simplex virus type 1 (HSV-1), binds to the CD80 promoter to suppress CD80 expression in antigen-presenting cells, leading to reduced T-cell function and protection. In contrast, overexpression of CD80 exacerbates corneal scarring (CS) in ocularly infected mice. In this study we tested the hypothesis that the absence of *ICP22* could increase disease severity.

**Methods:**

To test our hypothesis, BALB/c mice were ocularly infected after corneal scarification with a recombinant HSV-1 lacking the **ICP22** gene with its parental wild-type (WT) virus (KOS) as a control. Virus replication in the eye, CS, angiogenesis, latency, and reactivation between *ICP22* null virus and WT KOS were determined. In addition, expression of IL-2, IL-4, IFN-γ, IFN-α, granzyme A, granzyme B, and perforin by CD4 and CD8 T cells in corneas of infected mice on days 3, 5, 7, 10, 14, 21, and 28 postinfection were determined by flow cytometry.

**Results:**

We found similar levels of eye disease and angiogenesis in mice following corneal scarification and ocular infection with the *ICP22* null virus or parental WT virus despite reduced virus replication in the eye and reduced latency and reactivation in mice ocularly infected with *ICP22* null virus. The similar level of eye disease in *ICP22* null virus– and WT virus–infected mice correlated with expression of various proinflammatory cytokines that infiltrated the eye after HSV-1 infection.

**Conclusions:**

Our study identified a critical role for *ICP22* in HSV-1 pathogenicity and suggests that HSV-1–associated CS is more dependent on host immune responses to infection than to virus replication in the eye. Thus, HSV-1 as means of survival uses *ICP22* as a mechanism of immune escape that protects the host from increased pathology.

Herpes simplex virus type 1 (HSV-1) infection begins at the mucosal epithelium or at a break in the skin where a productive infection initiates within epithelial cells, and virus subsequently undergoes retrograde transport to sensory neurons.^1^ After infection is established in neurons, it becomes a persistent latent infection that is maintained for the life of the host.^2^ Approximately 70% to 90% of the US population is infected with either HSV-1 or HSV-2, while only 25% of the infected population shows clinical symptoms.^3^–^7^ Antiviral treatments available for herpes patients have a limited ability to suppress viral replication because once reactivation occurs, antiviral drugs cannot prevent the pathology.^8^ Therefore, understanding the basis of viral infectivity at genetic and cellular levels is of utmost importance.

Herpes stromal keratitis (HSK), also known as corneal scarring (CS),^9^,^10^ is a unique challenge to the infected host because it can evade initial antiviral immune responses mediated by interferons and other innate cell compartments.^11^ Primary infection and latency in the trigeminal ganglion (TG) lead to a robust adaptive immune response that is synchronized with profound reprogramming of the host immune response, including antigen exposure during reactivation, T-cell exhaustion, and differential cell infiltration, forming the basis of this study.^12^–^14^ Antigen exposure and recognition via the T-cell receptor (TCR) mediate events leading to T-cell activation, proliferation, and cytokine secretion, which promote or inhibit infection.^15^ Along with TCR binding to antigen-major histocompatibility complex (Ag-MHC) class II complexes on antigen-presenting cells (APCs), T cells require a second signal that is generated when CD28, CTLA-4, PD-1, and other T-cell markers bind to the costimulatory molecules CD80 (B7-1) or CD86 (B7-2) that are expressed on APCs.^16^,^17^ The costimulatory molecule CD80/86 drives T-cell activation and proliferation by binding to CD28.^18^,^19^ Recent studies^17^,^20^ have shown a role for PD-L1, which binds to CD28 and PD-1, in inhibiting T-cell activation and cytokine production.

We previously have shown that latency associated transcript (LAT) plays a vital role in generating dysfunctional T-cell responses in the TG of ocularly infected HSV-1 mice.^12^ We have found that increased LAT expression correlates with increased levels of PD1 and Tim-3 mRNAs, which are exhaustion markers expressed on anergic CD8^+^ T cells.^12^ We have also shown that CD8α^+^ dendritic cells (DCs), rather than CD8^+^ T cells, drive latency and reactivation, and that CD8^+^ T cells play a bystander role in maintaining latency in the TG of latently infected mice.^21^

To study the role of CD80 in T-cell stimulation, we constructed a recombinant virus with two copies of CD80 (HSV-CD80) in place of LAT and observed enhanced T-cell activation leading to more eye disease.^17^,^22^ Consistent with this idea and to determine the functional importance of CD80 in our model, we used a recombinant virus D22 that lacks *ICP22*.^23^,^24^ *ICP22* has a dual role, being required for virus replication^23^ and having an important role in downregulating CD80 expression after ocular HSV-1 infection.^22^ *ICP22* is required in vivo but its requirement in vitro is cell-line specific.^25^ This report extends our previous studies in which we have used in vivo and in vitro approaches to show that CD80 expression on DCs, but not on B cells, monocytes, macrophages, or T cells, is suppressed after primary HSV-1 infection in the eye.^22^ CD80 function is mediated by direct binding of HSV-1 *ICP22* to the CD80 promoter.^22^ Here we investigated the possible effect of *ICP22* absence on the kinetics of infiltration into the cornea of infected mice and the relationship of these infiltrates with eye disease.

Here, we compared D22, an *ICP22* null virus, to parental WT virus (KOS), and determined the effect of *ICP22* on the immunopathology of immune infiltrates into the cornea, eye disease, latency, reactivation, and viral replication. In the absence of *ICP22*, viral titers were significantly lower in the eyes of mice infected with *ICP22* null virus than in those infected with KOS virus. The level of latency and reactivation was also significantly lower in mice infected with *ICP22* null virus than in those infected with parental KOS virus, and PD-1 expression was downregulated in latently infected TG. The kinetics of immune cell infiltration into the cornea of *ICP22* null virus–infected mice was slower than in KOS virus infection. Thus, even though *ICP22* controlled viral replication and reactivation, upregulation of CD80 in *ICP22* null–infected mice could equalize immune cell infiltration into the eye and induce eye disease similar to that induced by WT virus. We speculate that *ICP22* regulates the interplay between viral replication and TCR signaling response once infection has established. In conclusion, our studies demonstrated a critical role for *ICP22* in reducing HSV-1–mediated immune pathology.

## Materials and Methods

### Viruses, Cells, and Mice

KOS and D22 recombinant viruses with an *ICP22* deletion have been described previously^23^ and were a generous gift from David Davido, University of Kansas. Vero cells were used to prepare virus stocks and virus titrations. Cells were grown in Eagle's minimal essential media supplemented with 5% fetal calf serum. Cells were typically passaged at 80% confluency and grown in a 37°C incubator with 5% CO_2_. Six-week-old female inbred BALB/c mice (The Jackson Laboratory, Bar Harbor, ME, USA) were used. All animal procedures adhered to the Association for Research in Vision and Ophthalmology (ARVO) Statement for the Use of Animals in Ophthalmic and Vision Research and according to institutional animal care and use guidelines. Animal research protocol was approved by the Institutional Animal Care and Use Committee of Cedars-Sinai Medical Center (Protocol No. 5030).

### Detection of Virus in Tears of Infected Mice

Tear films were collected from both eyes of 20 mice per group on days 1 to 7 postinfection (PI) by using a Dacron-tipped swab.^26^ Each swab was placed in 1 mL tissue culture medium, squeezed, and the amount of virus was determined by using a standard plaque assay on Vero cells.

### Monitoring Corneal Scarring and Angiogenesis

The severity of SK lesions in the corneas of mice was examined by slit-lamp biomicroscopy. Scoring was as follows: 0, normal cornea; 1, mild haze; 2, moderate opacity; 3, severe corneal opacity but iris visible; 4, opaque and corneal ulcer; 5, corneal rupture and necrotizing keratitis. The severity of angiogenesis was recorded by using a system in which a grade of 4 for a given quadrant of the circle represents a centripetal growth of 1.5 mm toward the corneal center. The score of the four quadrants of the eye was summed to derive the neovessel index (range, 0–16) for each eye at a given time point.^27^ Each cornea was examined and the mean ± SEM was calculated for each group.

### Ocular Infection and Isolation of Cornea for FACS

BALB/c mice were infected ocularly with 2 μL tissue culture media containing 2 × 10^5^ plaque forming units (PFU)/eye of the HSV-1 strains KOS or D22 (*ICP22* null) with corneal scarification. On days 3, 5, 7, 10, 14, 21, or 28 PI, infected mice were euthanized, and the corneas from each mouse were combined. Corneas were digested in a PBS solution containing collagenase type I (3 mg/mL; Sigma-Aldrich Corp., St. Louis, MO, USA) and incubated for 2 hours at 37°C with trituration approximately every 30 minutes as we have described previously.^28^ After washing the cell suspension, pelleted cells were resuspended in cell culture medium that was treated with Brefeldin A for 1 hour (cat. 420061; BioLegend, San Diego, CA, USA). Single cells were stained with anti-CD80, anti-CD4, anti-CD8, anti–IL-2, anti–IL-4, anti–granzyme B, anti–granzyme A, anti–IFN-α, anti–IFN-γ, anti-perforin, and anti-CD25 antibodies for 1 hour. All antibodies were purchased from BioLegend. Stained cells were washed two times with FACS buffer (1X PBS with 0.1% sodium azide), resuspended in 4% paraformaldehyde, and analyzed in a BD LSR II flow cytometer using BD FacsDiva Software (BD Biosciences, San Jose, CA, USA). Postexperiment data analysis was performed in FlowJo software (TreeStar). Experiments were repeated three times.

### RNA Extraction, cDNA Synthesis, and TaqMan RT-PCR

TG from individual mice were collected on day 28 PI, immersed in RNAlater RNA Stabilization Reagent (Thermo Fisher Scientific, Waltham, MA, USA), and stored at −80°C until processing. In some experiments, TG from uninfected mice of the same age as infected mice were collected as above. Total RNA extraction was conducted as we have described previously.^29^,^30^ Levels of LAT RNA from latent TG were determined by using a custom-made primer and probe set for LAT as follows: forward primer, 5′-GGGTGGGCTCGTGTTACAG-3′; reverse primer, 5′-GGACGGGTAAGTAACAGAGTCTCTA-3′; and probe, 5′-FAM-ACACCAGCCCGTTCTTT-3′ (amplicon length = 81 bp). The amplicon for the LAT primer set corresponds to LAT nucleotides 119553 to 119634. Relative LAT copy numbers were calculated by using standard curves generated from the plasmid pGem-LAT5317.

Levels of CD4, CD8, PD-1, and CD80 transcripts in TG were evaluated by using commercially available TaqMan Gene Expression Assays (Applied Biosystems, Foster City, CA, USA) with optimized primer and probe concentrations. Primer probe sets consisted of two unlabeled PCR primers and the FAM dye–labeled TaqMan MGB probe formulated into a single mixture. Additionally, all cellular amplicons included an intron-exon junction to eliminate signals from genomic DNA contamination. The assays used in this study were as follows: (1) CD4, ABI Mm00442754_m1 (amplicon length = 72 bp); (2) CD8 (α chain), ABI Mn01182108_m1 (amplicon length = 67 bp); (3) PD-1 (programmed death 1; also known as CD279), ABI Mm00435532_m1 (amplicon length = 65 bp); (4) CD80, ABI MM00711660_m1 (amplicon length = 117 bp); and (5) GAPDH used for normalization of transcripts, ABI Mm999999.15_G1 (amplicon length = 107 bp).

### Statistical Analysis

Protective parameters were plotted and analyzed for statistical significance by using Student's *t*-test, Fisher's exact test, 1-way ANOVA, or 2-way ANOVA with Bonferroni posttest with GraphPad (GraphPad, San Diego, CA, USA). Results were considered to be statistically significant if the *P* value was <0.05 (*), *P* < 0.01 (**), and *P* < 0.001 (***).

## Results

### Absence of *ICP22* Leads to Reduced Viral Replication in the Eyes of Ocularly Infected Mice

**Figure 1:**
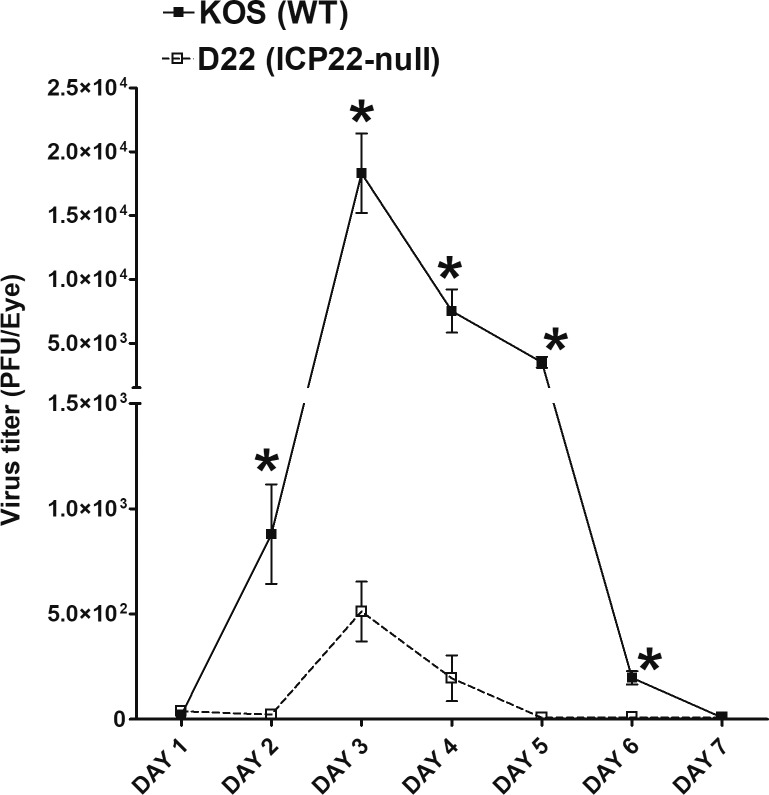
Viral titers in BALB/c mouse eyes following ocular infection with KOS strain of HSV-1 and D22 (ICP22 mutant) transgenic HSV-1 virus. Corneas from WT mice were scarified before ocular infection. They were then infected ocularly with 2 × 10^5^ PFU per eye of KOS or ICP22 null virus. The presence of infectious virus in the eyes of mice was monitored daily for 7 days by collecting tear films and quantifying virus, using standard plaque assays as described (see Materials and Methods). Each bar represents the mean ± SEM from 40 eyes for KOS-infected mice, and 40 eyes for ICP22 null–infected mice.

We previously have shown that HSV-1 suppresses CD80 activity in the cornea of ocularly infected mice and that HSV-1 *ICP22*, and no other HSV-1 gene, suppresses CD80 activity in vitro.^22^ In addition, significantly less CD11c and CD80 was expressed in the corneas of infected mice between days 1 and 7 PI than in corneas of mock-infected control animals. HSV-1 suppression of CD80 correlates directly with the presence of virus in the cornea of infected mice. Because we previously have compared WT virus–infected mice with mock-infected mice,^22^ here we investigated the effect of *ICP22* on HSV-1 infectivity.^23^,^24^ BALB/c mice were ocularly infected with 2 × 10^5^ PFU/eye of WT HSV-1 strain KOS or D22, an *ICP22* null virus in the KOS background with corneal scarification. Virus replication in the eye of ocularly infected mice was monitored daily by collecting tear films on days 1 to 7 PI. The presence of infectious virus was quantified by standard plaque assay, as described in Materials and Methods. We found that both parental KOS and D22 (*ICP22* null) viruses showed similar levels of infectious virus on day 1 PI, while mice infected with KOS had significantly higher virus titers on days 2 to 6 PI (Fig. 1, *P* < 0.0001). Virus titers peaked on day 3 PI and by day 7 PI, virus infectivity had declined for both viruses (Fig. 1). Consistent with our previous study,^23^ these results suggest that the absence of *ICP22* significantly hampers virus replication in the eye of infected mice and hastens viral clearance as compared to parental virus.

### Deficiency of *ICP22* Reduced Viral Latency and Explant Reactivation in *ICP22* Null Virus–Infected TG

**Figure 2:**
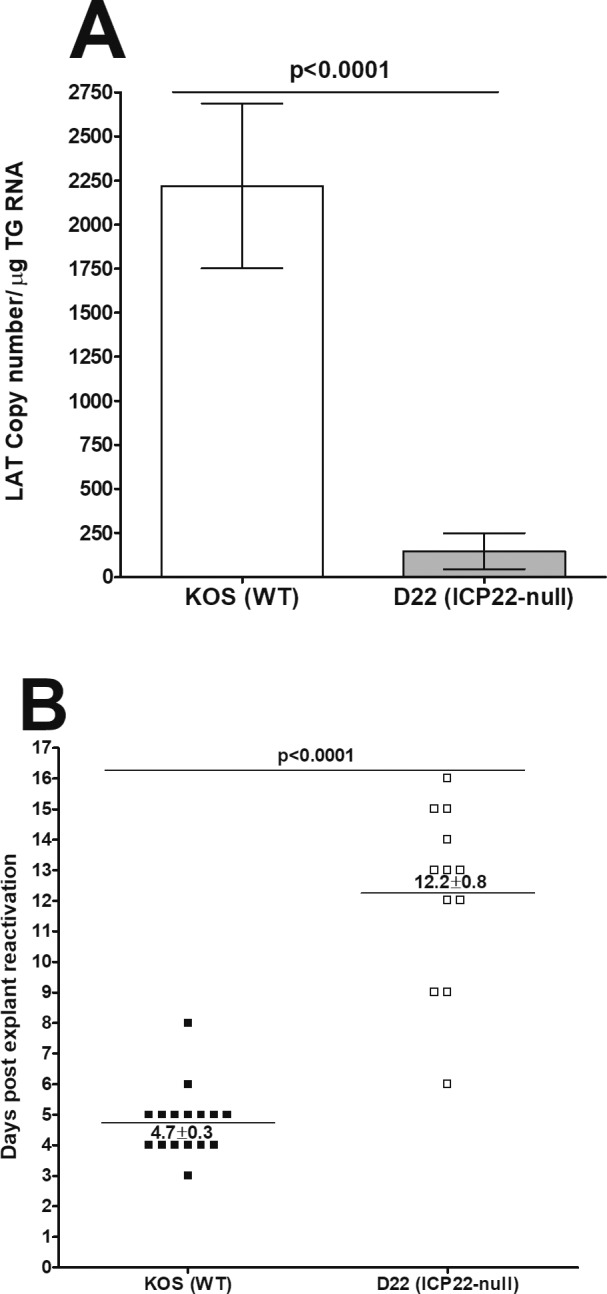
Level of latency and duration of explant reactivation following ocular infection of mice with avirulent KOS and ICP22 null viruses. Corneas from WT mice were scarified before ocular infection and infected ocularly with 2 × 10^5^ PFU per eye of KOS or ICP22 null virus as in Figure 1. On day 28 PI, TG from infected mice were harvested for RT-PCR and explant reactivation. (A) LAT RNA in latent TG. Quantitative RT-PCR was performed on each individual mouse TG. In each experiment, an estimated relative copy number of HSV-1 LAT was calculated by using standard curves generated from pGem5317. The plasmid template was serially diluted 10-fold such that 10 μL contained from 10^3^ to 10^11^ copies of LAT. Serial dilutions were then analyzed by TaqMan Real-time PCR with the same probe set. The copy number for each reaction was determined by comparing the normalized threshold cycle of each sample to the threshold cycle of the standard. GAPDH expression was used to normalize relative viral LAT RNA expression in the TG. Each bar represents the mean ± SEM from 18 TG for KOS-infected mice, and 16 TG for ICP22 null–infected mice. (B) Explant reactivation in latent TG. Individual TG from infected mice were incubated in 1.5 mL tissue culture media at 37°C and the presence of infectious virus was monitored at 20 days as described in the Material and Methods section. For each virus, 20 TG from 10 mice were used. The results are shown as the number of TG that reactivated daily. Each point represents the mean ± SEM from 16 TG for KOS and 12 TG for ICP22 null–infected mice. However, not all TG reactivated during the 20-day monitoring period: 4/20 KOS-infected TG and 8/20 ICP22 null–infected TG failed to reactivate. (A) LAT RNA. (B) Explant reactivation.

A major characteristic of HSV-1 infection is its ability to establish latency and frequent episodes of reactivation after latency.^2^,^13^,^14^ Thus, we sought to determine the effects of *ICP22* deletion on latency and reactivation following HSV-1 infection. To assess the effect of *ICP22* on establishment of latency, mice were ocularly infected with parental KOS or D22 (*ICP22* null) HSV-1 virus as described above. On day 28 PI, the amount of LAT transcript in latently infected individual TG was measured by qRT-PCR. Significantly higher levels of LAT mRNA were detected in the TG of KOS-infected mice than in D22 (*ICP22* null)–infected mice (*P* < 0.0001, Fig. 2A). These results suggest that the absence of *ICP22* is associated with decreased HSV-1 latency in the TG of latently infected mice.

To determine whether reduced LAT expression in TG of *ICP22* null–infected mice correlated with reduced latent virus reactivation, mice were ocularly challenged with KOS or D22 (*ICP22* null) virus. On day 28 PI, latently infected TG were harvested and the kinetics of virus reactivation was measured in the explanted TG. We observed significantly delayed reactivation in D22 (*ICP22* null)–infected mice (12.2 days) compared to KOS-infected mice (4.7 days, Fig. 2B). Reactivation levels in KOS- and D22 (*ICP22* null)–infected TG groups were statistically different (*P* < 0.0001, Fig. 2B). Slower virus reactivation in D22 is directly correlated with lower level of latency in mice infected with D22 virus than in KOS-infected mice. These results are consistent with a previous report showing that KOS-infected mice have more ocular viral replication and higher levels of latency than D22 (*ICP22* null virus)–infected mice.^23^ These results demonstrate that the absence of *ICP22* in the D22 virus reduced both latency and reactivation as compared to WT KOS-infected mice.

### Reduced Expression of PD-1, CD4, CD8, and CD80 mRNA Transcripts in the TG of D22 (*ICP22* Null) Latently Infected Mice

**Figure 3:**
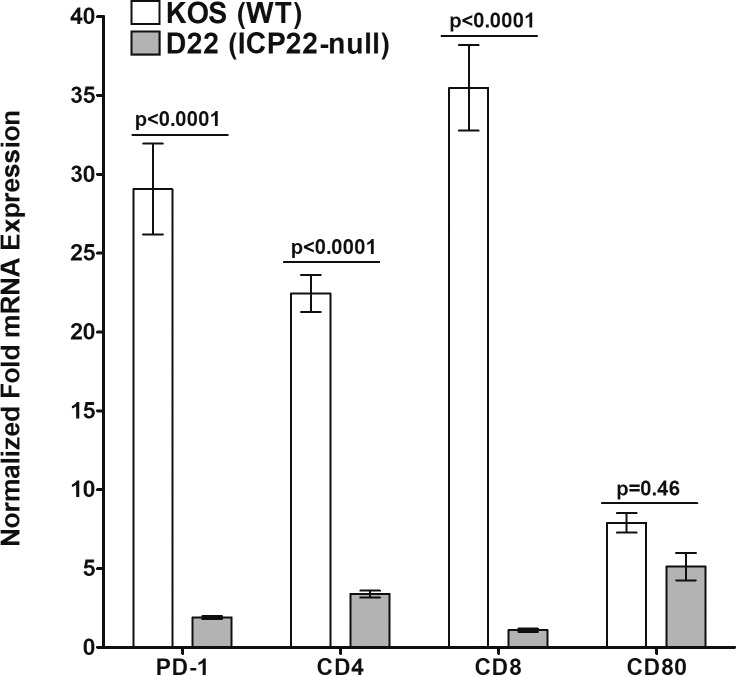
Quantitation of PD-1, CD4, CD8, and CD80 RNA transcripts in TG of latently infected BALB/c mice with avirulent parental KOS and ICP22 null HSV-1 virus. TG were collected from mice infected with KOS or ICP22 null HSV-1. Virus was harvested from TG on day 28 PI (after latency). Total RNA isolated from each TG was used to estimate the relative expression of each transcript in the TG of BALB/c mice. GAPDH expression was used to normalize the expression of each transcript in the TG of ocularly infected mice. For parental KOS, each bar represents the mean ± SEM from 12 TG, while for ICP22 null–infected mice, each bar represents the mean ± SEM from 13 TG.

We next determined whether the absence of *ICP22* has a functional impact on HSV-1–specific T cells in the TG. Exhaustion is a fundamental event in many viral infections and PD-1 transcript levels are a marker of T-cell exhaustion,^31^ and increased PD-1 expression correlates with increased HSV-1 latency in TG.^12^,^32^,^33^ To compare PD-1 transcript levels with T-cell function in latently infected TG, mice were infected with KOS or D22 (*ICP22* null) viruses as above. On day 28 PI, TG were isolated and levels of CD4, CD8, CD80, and PD-1 transcripts were quantitated by qRT-PCR. The results are presented as “fold” change in transcript level for each gene over its baseline mRNA level in TG from uninfected naive mice (Fig. 3). PD-1 transcripts were significantly higher in the TG of KOS-infected mice than in D22 (*ICP22* null)–infected TG (PD-1, *P* < 0.0001; Fig. 3). Levels of CD4 and CD8 T-cell transcripts in KOS-infected TG were also significantly higher than in TG of D22 (*ICP22* null)–infected mice (CD4 and CD8, *P* < 0.0001; Fig. 3). In contrast, CD80 expression was not statistically different between KOS- and *ICP22* null–infected mice (CD80, *P* = 0.46; Fig. 3). The similarity between the level of CD80 expression in TG of latently infected KOS and *ICP22* null viruses is expected, since previously we have reported that the suppression of CD80 by *ICP22* is dependent on the presence of active virus replication.^22^ These results show a direct correlation between lower replication in the eye, lower latency, lower reactivation, lower PD-1, and lower T-cell transcripts in D22 (*ICP22* null) virus infections.

### Reduced Replication of D22 Virus Does Not Correlate With Eye Disease and Angiogenesis

**Figure 4:**
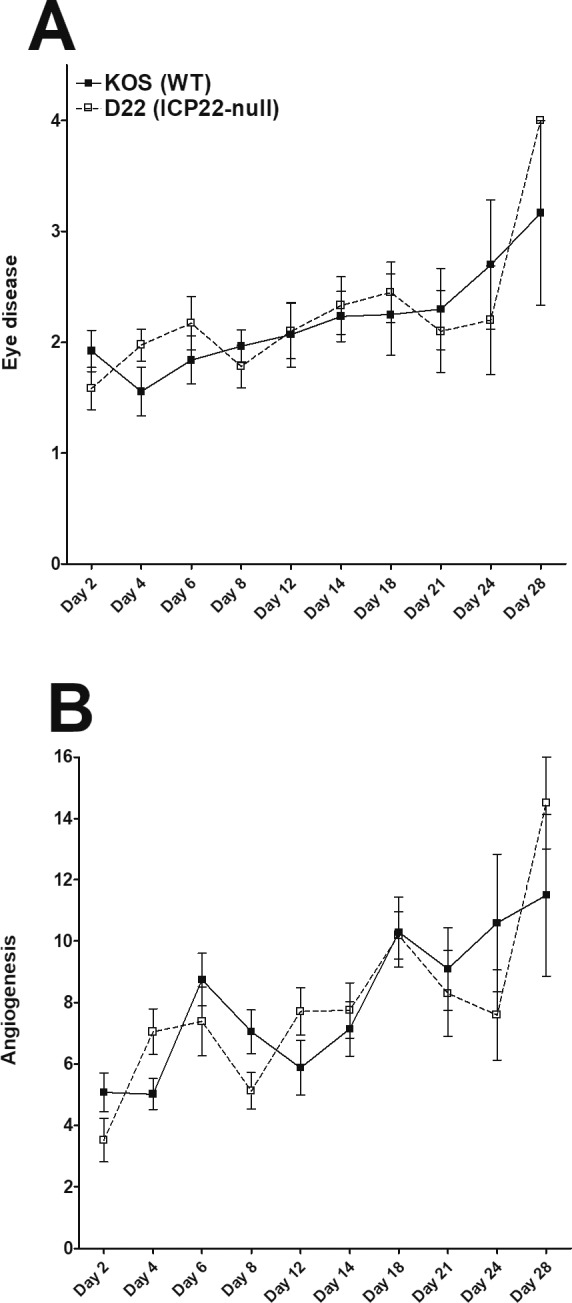
Loss of ICP22 contributes to SK severity. Corneas from WT BALB/c mice were scarified before ocular infection and then were infected with 2 × 10^5^ PFU per eye of KOS (WT ICP22) or ICP22 null (mutant deleted ICP22) virus as described in Materials and Methods. SK lesion severity in the mouse corneas was examined by slit-lamp biomicroscopy. Severity was scored as follows: 0, normal cornea; 1, mild haze; 2, moderate opacity; 3, severe corneal opacity but iris visible; 4, opaque and cornea ulcer; 5, corneal rupture and necrotizing keratitis. (A) Kinetics of SK severity is shown on days 2, 4, 6, 8, 12, 14, 18, 21, 24, and 28 PI. Angiogenesis severity was recorded by using a system in which a grade of 4 for a given quadrant of the circle represents a centripetal growth of 1.5 mm toward the corneal center. Scores of the four quadrants of the eye were then summed to derive the neovessel index (range, 0–16) for each eye at a given time point. (B) Kinetics of angiogenesis severity is shown on days 2, 4, 6, 8, 12, 14, 18, 21, 24, and 28 PI. Experiments were repeated three times and each bar represents the mean ± SEM from 20 eyes for KOS-infected mice, and 20 eyes for ICP22 null–infected mice.

Immune cell infiltration following ocular HSV-1 infection contributes to the pathologic immune response that characterizes stromal keratitis (SK) and corneal neovascularization.^27^ To determine the effect of *ICP22* deletion on disease progression and angiogenesis after HSV-1 infection, BALB/c mice were infected with either KOS or D22 (*ICP22* null) virus as described in Materials and Methods, and eye disease was compared at various time points to monitor corneal damage. The kinetics of eye disease and angiogenesis on days 2, 4, 6, 8, 12, 14, 18, 21, 24, and 28 PI are shown in Figure 4. The results showed no significant differences in eye disease (Fig. 4A) or angiogenesis (Fig. 4B) in mice infected with either WT KOS or D22 (*ICP22* null) virus. Although mice challenged with *ICP22* null virus showed a slight increase in SK (Fig. 4A) and angiogenesis (Fig. 4B) over the time course, they did not differ statistically from those infected with parental KOS virus. We also observed significant reductions in viral titers, latency, and reactivation in D22 (*ICP22* null)–infected groups compared to KOS groups (Figs. 1, 2), but the impact of such responses did not affect eye disease and angiogenesis in D22 (*ICP22* null)–infected mice compared with KOS-infected mice. Therefore, we hypothesize that deletion of *ICP22* induces CD80 promoter activity to enhance T-cell responses. As a result, effector T-cell responses in D22 (*ICP22* null)–infected mice rise to the level of KOS-infected mice and induce pathology similar to that seen in KOS infection, but do not significantly increase pathology when compared to KOS infection owing to reduced virus infectivity. Our results are similar to the previous study in which following ocular HSV-1 infection, angiogenesis was detected as early as 24 hours PI and peaked by day 14 PI.^34^

### D22 (*ICP22* Null) Virus and WT KOS Have Similar Levels of T-Cell Infiltration Despite Poor Replication of D22 in Infected Mice

CD4 and CD8 have both been implicated in enhancement of eye disease in ocularly infected mice.^33^,^35^ To further explore the effect of *ICP22* on CD80 activation in vivo and subsequent eye disease, mice were ocularly infected with 2 × 10^5^ PFU/eye of KOS or D22 (*ICP22* null) virus as above. The extent of ocular inflammation in single cell suspensions of corneas from KOS- and D22 (*ICP22* null virus)–infected mice was compared on days 3, 5, 7, 10, 14, 21, and 28 PI by flow cytometry as described in Materials and Methods.

**Figure 5:**
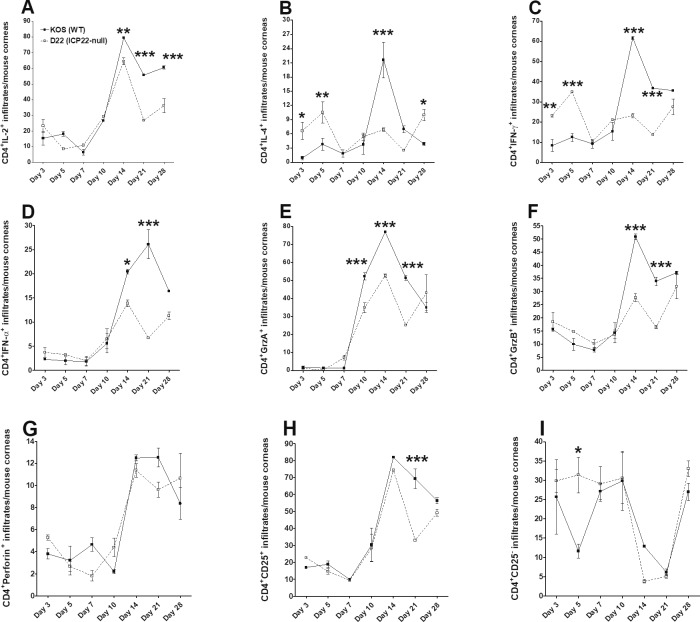
Effect of ICP22 on CD4^+^ T cell infiltration by flow cytometry. Effect of HSV-1 infection on CD4^+^ T cells and cytokine expression profiles in corneas of infected mice. BALB/c mice were infected in both eyes with 2 × 10^5^ PFU/eye of KOS parental virus or ICP22 null virus as described in Materials and Methods. Corneas from infected mice were isolated on days 3, 5, 7, 10, 14, 21, and 28 PI. Corneas from each mouse were harvested, digested with collagenase, and the cell suspension was stained with anti-CD4, anti–IL-2, anti–IL-4, anti–granzyme B, anti–granzyme A, anti–IFN-α, anti–IFN-γ, anti-perforin, and anti-CD25 antibodies before flow cytometry as described in Materials and Methods. (A) Percentage of CD4^+^IL-2^+^ T cells. (B) Percentage of CD4^+^IL-4^+^ T cells. (C) Percentage of CD4^+^IFN-γ^+^ T cells. (D) Percentage of CD4^+^IFN-α^+^ T cells. (E) Percentage of CD4^+^GrzA^+^ T cells. (F) Percentage of CD4^+^GrzB^+^ T cells. (G) Percentage of CD4^+^perforin^+^ T cells. (H) Percentage of CD4^+^CD25^+^ T cells (CD4^+^ T-regs). (I) Percentage of CD4^+^CD25^−^ T cells (CD4^+^ effector T cells). The experiments were done with N = 5 mice per time point. Experiments were repeated three times.

The CD4^+^IL2^+^ and CD4^+^IFN-γ^+^ cells showed time-dependent differences in subpopulation mean percentages between D22 (*ICP22* null)– and KOS virus–infected groups (Figs. 5A, 5C). The peak of CD4^+^IL-2^+^ responses following KOS infection on day 14 PI was greater than responses to D22 (*ICP22* null) virus infection (79.4% vs. 64.5%, *P* < 0.01) and was reduced on day 28 PI (60.5% vs. 36.2%, *P* < 0.001; Fig. 5A). In contrast, while CD4^+^IFN-γ^+^ responses were greater following KOS infection than D22 (*ICP22* null) virus infection on day 14 PI (61.7% vs. 23.2%, *P* < 0.001), the differences between these viruses were not statistically significant on day 28 PI (35.6% vs. 27.5%, *P* > 0.05; Fig. 5C).

The CD4^+^IL-4^+^ cytokine response following KOS virus infection was elevated on day 14 PI, compared to D22 (*ICP22* null) virus infection (21.6% vs. 6.9%, *P* < 0.001), but differences between these groups were not significant on day 28 PI (3.9% vs. 10%, *P* > 0.05; Fig. 5B). Interestingly, mean cytokine expression levels in the D22 (*ICP22* null)–infected group usually peaked around day 28 PI with mean differences that did not differ significantly from the KOS-infected group for the following infiltrates: CD4^+^IFN-α^+^ (11.3%), CD4^+^GrzA^+^(27.5%), CD4^+^GrzB^+^ (32%), CD4^+^perforin^+^ (10%), CD4^+^CD25^+^ (49%), and CD4^+^CD25^−^ (33%) (Figs. 5D–I). The kinetics of CD4^+^ T-cell expression showed that while KOS usually had a higher peak than D22 (*ICP22* null) on day 14 PI when lesion development is maximum, cytokine levels in *ICP22* null–infected corneal cells increased to levels that did not significantly differ from KOS infection on day 28 PI. Thus, loss of HSV-1 *ICP22* appears to delay the peak response of CD4^+^ immune cell infiltrates and matched effector cell populations in KOS infection, resulting in no differences in eye disease pathology between *ICP22* null– and KOS-infected mice groups.

**Figure 6:**
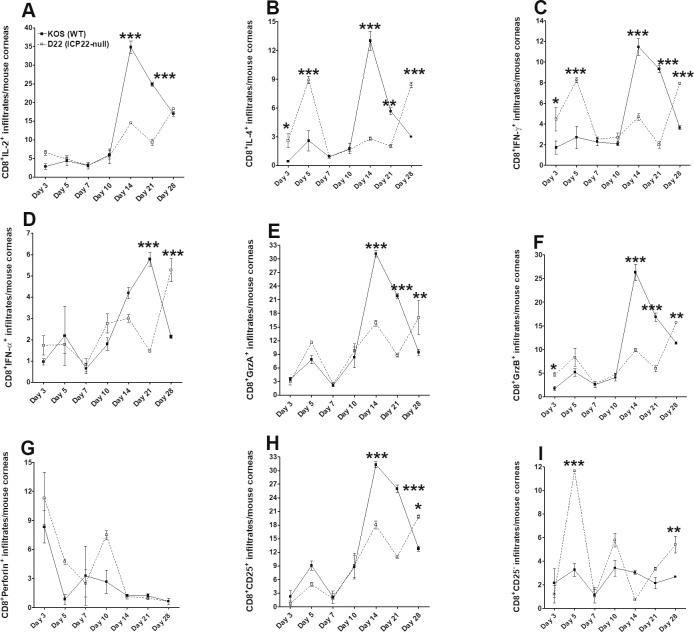
Effect of ICP22 on CD8^+^ T-cell infiltration by flow cytometry. Effect of HSV-1 infection on CD8^+^ T cells and cytokine expression profiles in corneas of infected mice. BALB/c mice were infected in both eyes with 2 × 10^5^ PFU/eye of KOS parental virus or ICP22 null virus as described in Materials and Methods. Corneas from infected mice were isolated on days 3, 5, 7, 10, 14, 21, and 28 PI. Corneas from each mouse were harvested, digested with collagenase, and the cell suspension was stained with anti-CD8, anti–IL-2, anti–IL-4, anti–granzyme B, anti–granzyme A, anti–IFN-α, anti–IFN-γ, anti-perforin, and anti-CD25 antibodies before flow cytometry as described in Materials and Methods. (A) Percentage of CD8^+^IL-2^+^ T cells. (B) Percentage of CD8^+^IL-4^+^ T cells. (C) Percentage of CD8^+^IFN-γ^+^ T cells. (D) Percentage of CD8^+^IFN-α^+^ T cells. (E) Percentage of CD8^+^GrzA^+^ T cells. (F) Percentage of CD8^+^GrzB^+^ T cells. (G) Percentage of CD8^+^perforin^+^ T cells. (H) Percentage of CD8^+^CD25^+^ T cells (CD8^+^ T-regs). (I) Percentage of CD8^+^CD25^−^ T cells (CD8^+^ effector T cells). The experiments were done with N = 5 mice per time point. Experiments were repeated three times.

We also looked at the CD8^+^ T-cell immune response and found that on day 28 PI, mean cytokine levels associated with *ICP22* null virus infection were all greater than or equal to mean cytokine levels in KOS virus infection. At day 28 PI, the immune response was significantly greater in *ICP22* null virus infection than in KOS virus infection for the following immune infiltrates: CD8^+^IL-4^+^ (8.4% vs. 3.0%, *P* < 0.001; Fig. 6B), CD8^+^GrzB^+^ (15.7% vs. 11.3%, *P* < 0.01; Fig. 6F), CD8^+^IFN-γ^+^ (7.9% vs. 3.7%, *P* < 0.001; Fig. 6C), CD8^+^IFN-α^+^ (5.3% vs. 2.1%, *P* < 0.001; Fig. 6D), CD8^+^GrzA^+^ (17.1% vs. 9.4%, *P* < 0.01; Fig. 6E), and CD8^+^CD25^+^ (19.8% vs. 12.8%, *P* < 0.05; Fig. 6H). Interestingly, following D22 (*ICP22* null) infection, cytokine levels increased to equal those following KOS infection for the following infiltrates: CD8^+^IL-2^+^ (18.3% vs. 16.9%, Fig. 6A), CD8^+^perforin^+^ (0.6% vs. 0.6%, Fig. 6G), and CD8^+^CD25^−^ (5.4% vs. 2.7%, Fig. 6I). In fact, following *ICP22* null virus infection, CD4^+^perforin^+^ and CD8^+^perforin^+^ expression levels were similar to those following KOS virus infection at all time points measured.

## Discussion

This report extends our previous findings and further explored the importance of *ICP22* and CD80 in HSV-1–induced immunopathology. We studied the role of *ICP22* after HSV-1 infection in eye disease, viral replication, latency, reactivation, and immune cell infiltrates into the eye. TCR signaling has been investigated thoroughly and it is known to be an intricate system that involves both costimulatory and coinhibitory receptor signaling.^36^ Therefore, TCR signaling is very important in T-cell function and fate. For example, TCR-mediated T-cell activation in the absence of costimulation is known to render T cells inactive, a process termed T-cell anergy. Thus, costimulation is a key decision point to determine if antigen exposure to T cells will lead to activation or anergy. Our previous findings have shown that HSV-CD80 infection exacerbates corneal scarring.^22^ We also have demonstrated that the suppression of CD80 in murine DCs, both in vivo and in vitro, is mediated by *ICP22* binding to the CD80 promoter, resulting in downregulation of CD80 transcript and protein expression in APCs.^22^ From our published *ICP22*-CD80 functional studies, we were intrigued by the hypothesis to explore HSV-1–induced CS in vivo using *ICP22* null virus infection.

In this study, we evaluated the role of *ICP22* by using a recombinant virus lacking *ICP22* and observed less viral replication, less latency, and less reactivation than in the parental virus model of KOS. The results of this study were similar to published studies showing that *ICP22* deletion significantly reduces viral replication and decreases the ability of virus to establish latent infection in the TG of HSV-1–infected mice.^37^ PD-1 is overexpressed on exhausted T cells and is inducibly expressed on B cells and activated monocytes.^19^ In a mouse model of HSK, PD-L1 expression has been reported to be upregulated on CD11b^+^ macrophages and on CD4^+^ T cells in draining lymph nodes and inflamed corneas after infection.^38^ Studies of human immunodeficiency virus, hepatitis C virus, and hepatitis B virus have shown that immune responses can be restored by PD1-PDL1 blockade.^39^ In a lymphocytic choriomeningitis virus (LCMV) model, PD1 expression increases on activated T cells and is eventually reduced with virus clearance.^40^ We saw less PD-1 expression in TG of *ICP22* null–infected mice than with KOS infection, which we hypothesize led to more T-cell activation and an increased trend in disease pathology on day 28 PI.

Another study using the LCMV model has found that although CD8^+^ T cells are known to clear virus from the infected site, there is a need to mediate homeostasis between CD8^+^ T-cell numbers and CD8^+^ T-cell exhaustion.^41^ The authors^41^ record more mortality in mice having a greater number of CD8^+^ T cells, leading to more perforin secretion and increased immunopathology. In our study, we found similar levels of perforin secretion by CD8^+^ T cells in KOS and D22 (*ICP22* null) mouse groups on day 10 PI, but perforin levels declined with similar amount of secretions in both infected groups. We found significantly more total CD8^+^ T cells, but no difference in total CD4^+^ T cell frequency, after HSV-1 infection on day 14 PI and day 21 PI (data not shown). Moreover, as reported previously, CD4^+^ T_H_1 cells are the principle orchestrators of HSK in the mouse model, whereas CD4^+^CD25^+^ Treg cells play a protective role against eye disease.^42^ We measured the levels of IFN-γ secretion by T_H_1 cells and also measured CD4^+^ Treg in corneas and found that the levels of IFN-γ secretion by T_H_1 cells and CD4^+^CD25^+^ T cells matched in both the infected groups on day 28 PI. Granzyme B secretion by CD8^+^ T cells is known for its cytolytic effect^43^,^44^ and was significantly higher on day 28 PI in *ICP22* null–infected group, and also the CD4^+^CD25^−^ effector cell population showed similar kinetics of expression in both KOS- and *ICP22* null–infected groups. Considering all these factors, this might explain why we observed similar levels of eye disease in both KOS- and D22 (*ICP22* null virus)–infected groups.

We hypothesized that enhanced CD80 expression would result in more eye disease in D22 (*ICP22* null)–infected mice despite the fact that we observed reduced virus replication in mice infected with *ICP22* null virus. We have reported previously that the suppression of CD80 by *ICP22* is dependent on the presence of active virus replication.^22^ Therefore, the lack of significant differences between the levels of CD80 expression in TG of latently infected KOS and *ICP22* null viruses is not unexpected. Even though mice infected with *ICP22* null virus display low viral replication, reduced latency, and reduced reactivation, they develop eye disease similar to that seen in KOS-infected mice. The similar influx of immune cell infiltrates into the cornea of *ICP22* null–infected mice in spite of lower viral load is probably the reason why *ICP22* null and KOS display similar levels of eye disease. Therefore, we speculate that despite reduced virus replication, the robust immune response generated by *ICP22* null infection caused equal damage to the corneas of infected mice as did infection with WT virus, even though WT virus has higher virus infectivity. Previous studies^23^,^24^ have shown that in the absence of *ICP22* virus replication is reduced in vitro and in vivo. In contrast to this, previously it has been shown that recombinant HSV-1 with a single mutation in amino acid 34 or 116 of *ICP22* has reduced eye disease, compared with parental control virus.^45^,^46^ This discrepancy between our results and previous studies is possibly due to the fact that, while mutation in amino acid 34 or 116 of *ICP22* affected virus replication and pathology, it did not eliminate its binding to CD80 promoter and thus still did suppress the immune response in the cornea of infected mice. However, in this study, in the absence of *ICP22*, there was no suppression of CD80 by *ICP22* and thus we observed more eye disease due to higher immune infiltrates in the cornea of mice infected with *ICP22* null virus than with parental control virus.

Taken together, our current findings confirm our previous in vitro results, which show that *ICP22* can suppress CD80,^22^ and extend these findings to an in vivo model by showing that suppression of CD80 by *ICP22* dampens the immune response, thereby protecting the virus and the host from immune-mediated pathology.

## References

[1] Roizman B, Whitley RJ (2013). An inquiry into the molecular basis of HSV latency and reactivation. *Annu Rev Microbiol*.

[2] Phelan D, Barrozo ER, Bloom DC (2017). HSV1 latent transcription and non-coding RNA: a critical retrospective. *J Neuroimmunol*.

[3] Wilhelmus KR, Dawson CR, Barron BA (1996). Risk factors for herpes simplex virus epithelial keratitis recurring during treatment of stromal keratitis or iridocyclitis: Herpetic Eye Disease Study Group. *Br J Ophthalmol*.

[4] Liesegang TJ (2001). Herpes simplex virus epidemiology and ocular importance. *Cornea*.

[5] Roberts CM, Pfister JR, Spear SJ (2003). Increasing proportion of herpes simplex virus type 1 as a cause of genital herpes infection in college students. *Sex Transm Dis*.

[6] Auslander BA, Biro FM, Rosenthal SL (2005). Genital herpes in adolescents. *Semin Pediatr Infect Dis*.

[7] Singh AE, Romanowski B, Wong T (2005). Herpes simplex virus seroprevalence and risk factors in 2 Canadian sexually transmitted disease clinics. *Sex Transm Dis*.

[8] Whitley R, Baines J (2018). Clinical management of herpes simplex virus infections: past, present, and future. *F1000Res*.

[9] Koelle DM, Ghiasi H (2005). Prospects for developing an effective vaccine against ocular herpes simplex virus infection. *Curr Eye Res*.

[10] Koujah L, Suryawanshi RK, Shukla D (2019). Pathological processes activated by herpes simplex virus-1 (HSV-1) infection in the cornea. *Cell Mol Life Sci*.

[11] Vandevenne P, Sadzot-Delvaux C, Piette J (2010). Innate immune response and viral interference strategies developed by human herpesviruses. *Biochem Pharmacol*.

[12] Allen SJ, Hamrah P, Gate DM (2011). The role of LAT in increased CD8+ T cell exhaustion in trigeminal ganglia of mice latently infected with herpes simplex virus type 1. *J Virol*.

[13] Wechsler SL, Nesburn AB, Watson R, Slanina S, Ghiasi H (1988). Fine mapping of the major latency-related RNA of herpes simplex virus type 1 in humans. *J Gen Virol*.

[14] Wechsler SL, Nesburn AB, Watson R, Slanina SM, Ghiasi H (1988). Fine mapping of the latency-related gene of herpes simplex virus type 1: alternative splicing produces distinct latency-related RNAs containing open reading frames. *J Virol*.

[15] Dimeloe S, Burgener AV, Grahlert J, Hess C (2017). T-cell metabolism governing activation, proliferation and differentiation; a modular view. *Immunology*.

[16] Bertram EM, Dawicki W, Watts TH (2004). Role of T cell costimulation in anti-viral immunity. *Semin Immunol*.

[17] Mott KR, Allen SJ, Zandian M (2014). Inclusion of CD80 in HSV targets the recombinant virus to PD-L1 on DCs and allows productive infection and robust immune responses. *PLoS One*.

[18] Borriello F, Sethna MP, Boyd SD (1997). B7-1 and B7-2 have overlapping, critical roles in immunoglobulin class switching and germinal center formation. *Immunity*.

[19] Sharpe AH, Freeman GJ (2002). The B7-CD28 superfamily. *Nat Rev Immunol*.

[20] Butte MJ, Keir ME, Phamduy TB, Sharpe AH, Freeman GJ (2007). Programmed death-1 ligand 1 interacts specifically with the B7-1 costimulatory molecule to inhibit T cell responses. *Immunity*.

[21] Mott KR, Gate D, Matundan HH, Ghiasi YN, Town T, Ghiasi H (2016). CD8+ T cells play a bystander role in mice latently infected with herpes simplex virus 1. *J Virol*.

[22] Matundan H, Ghiasi H (2019). Herpes simplex virus 1 *ICP22* suppresses CD80 expression by murine dendritic cells. *J Virol*.

[23] Mostafa HH, Davido DJ (2013). Herpes simplex virus 1 *ICP22* but not US 1.5 is required for efficient acute replication in mice and VICE domain formation. *J Virol*.

[24] Rice SA, Davido DJ (2013). HSV-1 *ICP22* hijacking host nuclear functions to enhance viral infection. *Future Microbiol*.

[25] Sears AE, Halliburton IW, Meignier B, Silver S, Roizman B (1985). Herpes simplex virus 1 mutant deleted in the alpha 22 gene: growth and gene expression in permissive and restrictive cells and establishment of latency in mice. *J Virol*.

[26] Ghiasi H, Bahri S, Nesburn AB, Wechsler SL (1995). Protection against herpes simplex virus-induced eye disease after vaccination with seven individually expressed herpes simplex virus 1 glycoproteins. *Invest Ophthalmol Vis Sci*.

[27] Jaggi U, Varanasi SK, Bhela S, Rouse BT (2018). On the role of retinoic acid in virus induced inflammatory response in cornea. *Microbes Infect*.

[28] Mott KR, UnderHill D, Wechsler SL, Ghiasi H (2008). Lymphoid-related CD11c+CD8a+ dendritic cells are involved in enhancing HSV-1 latency. *J Virol*.

[29] Mott KR, Perng GC, Osorio Y, Kousoulas KG, Ghiasi H (2007). A recombinant herpes simplex virus type 1 expressing two additional copies of gK is more pathogenic than wild-type virus in two different strains of mice. *J Virol*.

[30] Mott KR, Osorio Y, Brown DJ (2007). The corneas of naive mice contain both CD4+ and CD8+ T cells. *Mol Vis*.

[31] Ahn E, Araki K, Hashimoto M (2018). Role of PD-1 during effector CD8 T cell differentiation. *Proc Natl Acad Sci U S A*.

[32] Mott KR, Allen SJ, Zandian M (2014). CD8a dendritic cells drive establishment of HSV-1 latency. *PLoS One*.

[33] Mott KR, Bresee CJ, Allen SJ, BenMohamed L, Wechsler SL, Ghiasi H (2009). Level of herpes simplex virus type 1 latency correlates with severity of corneal scarring and exhaustion of CD8+ T cells in trigeminal ganglia of latently infected mice. *J Virol*.

[34] Biswas PS, Rouse BT (2005). Early events in HSV keratitis—setting the stage for a blinding disease. *Microbes Infect*.

[35] Suvas S, Kim B, Rouse BT (2008). Homeostatic expansion of CD4(+) T cells upregulates VLA-4 and exacerbates HSV-induced corneal immunopathology. *Microbes Infect*.

[36] Gaud G, Lesourne R, Love PE (2018). Regulatory mechanisms in T cell receptor signalling. *Nat Rev Immunol*.

[37] Poffenberger KL, Idowu AD, Fraser-Smith EB, Raichlen PE, Herman RC (1994). A herpes simplex virus type 1 *ICP22* deletion mutant is altered for virulence and latency in vivo. *Acta Microbiol Immunol Hung*.

[38] Jun H, Seo SK, Jeong HY (2005). B7-H1 (CD274) inhibits the development of herpetic stromal keratitis (HSK). *FEBS Lett*.

[39] Sharpe AH, Wherry EJ, Ahmed R, Freeman GJ (2007). The function of programmed cell death 1 and its ligands in regulating autoimmunity and infection. *Nat Immunol*.

[40] Barber DL, Wherry EJ, Masopust D (2006). Restoring function in exhausted CD8 T cells during chronic viral infection. *Nature*.

[41] Christiaansen AF, Schmidt ME, Hartwig SM, Varga SM (2017). Host genetics play a critical role in controlling CD8 T cell function and lethal immunopathology during chronic viral infection. *PLoS Pathog*.

[42] Suvas S, Azkur AK, Kim BS, Kumaraguru U, Rouse BT (2004). CD4+CD25+ regulatory T cells control the severity of viral immunoinflammatory lesions. *J Immunol*.

[43] Metkar SS, Wang B, Aguilar-Santelises M (2002). Cytotoxic cell granule-mediated apoptosis: perforin delivers granzyme B-serglycin complexes into target cells without plasma membrane pore formation. *Immunity*.

[44] Trapani JA, Smyth MJ (2002). Functional significance of the perforin/granzyme cell death pathway. *Nat Rev Immunol*.

[45] Brandt CR, Kolb AW (2003). Tyrosine 116 of the herpes simplex virus type 1 IEalpha22 protein is an ocular virulence determinant and potential phosphorylation site. *Invest Ophthalmol Vis Sci*.

[46] Brandt CR, Kolb AW, Shah DD (2003). Multiple determinants contribute to the virulence of HSV ocular and CNS infection and identification of serine 34 of the US1 gene as an ocular disease determinant. *Invest Ophthalmol Vis Sci*.

